# Association of Biochemical and Hematological Parameters With Enteric Fever Infection at the Dschang Regional Annex Hospital, Cameroon: A Cross-Sectional Study

**DOI:** 10.7759/cureus.40498

**Published:** 2023-06-16

**Authors:** Tito Aloys N Etouke, Georges Ful Kuh, Vanessa Linda Nzesseu, Boris Emmanuel D Gomseu, Jean-De-Dieu Tamokou, Jean Paul Dzoyem

**Affiliations:** 1 Department of Biochemistry, University of Dschang, Dschang, CMR

**Keywords:** cross-sectional study, hematological profile, biochemical parameters, association, enteric fever

## Abstract

Background

Enteric fever is a systemic infection in humans caused by the Gram-negative bacilli*Salmonella enterica* serovars Typhi and Paratyphi*.* Although the diagnosis typically involves the isolation of *Salmonella enterica* serovars, it is often determined based on laboratory findings and clinical observations. However, due to the wide variety and the non-specific character of clinical features, making a definitive diagnosis presents numerous challenges. Therefore, the aim of this study was to find the predictive hematological and biochemical parameters which would serve in the diagnosis, prognosis, and treatment of typhoid fever cases.

Methodology

A cross-sectional study was conducted from November 2020 to September 2021 on1076consented volunteerparticipants. Stool culture and identification tests enabled us to distinguish three groups including 423 *Salmonella *Typhi positive patients, 115 *S. *Paratyphi positive patients, and 538 *Salmonella *negative participants. Biochemical and hematological parameters were evaluated using standard methods from commercial kits and Sysmex KX-21N automated hematology analyzer, respectively. A multiple logistic regression analysis was performed to identify the validity of the hematological and biochemical characteristics for enteric fever diagnosis.

Results

Multiple logistic regression showed hyper creatininemia, hypoalbuminemia, hyper total proteinemia, hyper alkaline phosphatase (ALP), hyper alanine aminotransferase (ALT), hyper total bilirubinemia, hyper conjugated bilirubinemia, hyper triglyceridemia, hyper C-reactive protein (CRP), leukopenia, thrombocytopenia, lymphopenia, monocytopenia, low hemoglobin, low hematocrit, low mean corpuscular volume (MCV), low mean corpuscular hemoglobin (MCH), low platelet, low platelet crit level, high platelet distribution width (PDW) level, high erythrocyte sedimentation rate 1 (ESR1) level as significant biological abnormalities associated (odds ratio {OR} > 1; p < 0.05) with enteric fever infection. Similarly, hyper ESR2 was an independent predictor (OR > 1; p < 0.05) of *S. *Typhi infection. However, a negative and significant association (OR < 1; p < 0.05) was recorded between enteric fever infection and high mean platelet volume (MPV).

Conclusion

Overall the results of the biochemical and hematological profiles can serve as potential diagnostic markers for typhoid fever. These markers can also be useful in the appropriate management of those with enteric fever, preventing severity and limiting outcomes of mortality.

## Introduction

Enteric fever is a disease caused by ingestion of the Gram-negative bacilli *Salmonella enterica* serovar Typhi and *Salmonella enterica* serovar Paratyphi (*S.* Typhi and *S.* Paratyphi) through contaminated food and water. The most common symptoms of enteric fever are nausea, fever, abdominal pain, and vomiting. It is a common infectious disease in industrialized and developing countries [1]. About 11-20 million people are affected by typhoid fever each year, with 128,000-161,000 deaths globally, mostly in low-and middle-income countries whereas 6 million cases are caused by paratyphoid fever with 54,000 deaths each year worldwide [2]. The disease is endemic in many developing countries, especially in the Indian subcontinent, South and Central America, and Africa, with an annual crude incidence estimate of 130 per 100,000 person-years of observation [3]. Enteric fever remains one of the major public health and economic problems in developing countries with 2.6 times higher incidence of typhoid fever than the overall incidence in middle-income countries. Furthermore, due to poor access to drinking water and sanitation facilities, typhoid fever is a serious health problem in Cameroon [4].

The gold standard test, such as isolation of the bacterium by blood culture, stool culture, or uroculture is not readily available in hospitals and clinics in a resource-limited country like Cameroon. Additionally, self-medication and abuse of antibiotics in the community often hinder the isolation of this bacterium through blood culture, stool culture, uroculture, and bone marrow culture, an invasive and difficult method. Hence, a serological test is widely used for the diagnosis of typhoid and paratyphoid fevers in most developing country laboratories. Unfortunately, the existing serodiagnostic assays such as Widal and Typhidot lack the required analytical sensitivity and specificity to diagnose typhoid fevers in disease-ravaging zones. Furthermore, requested serological assays are often misinterpreted due to the lack of appropriate control agglutination assays and deficient knowledge of assays’ basic principles. 

*S. *Typhi and Paratyphi cause localized infection of the gastrointestinal tract but can also multiply in the reticulo-endothelial cells and macrophages resulting in systemic infection. Hence, they affect one or more major organs of the body culminating in a wide range of organ infections such as hepatitis, gastroenteritis, myocarditis, endocarditis, and glomerulonephritis with or without associated acute renal failure or tubular necrosis [5]. This extended and severe infection may be multifactorial, involving endotoxin, local inflammatory, and/or immune reactions of the host. The development of encephalopathy, hemophagocytosis, anemia, leucopenia, gastrointestinal perforations, and acute respiratory distress syndrome (ARDS) are rare but represent serious complications of typhoid fever [6]. Hence, the incorporation of biochemical and hematological parameters is a source of potent biomarkers that would identify enteric fever and may also be utilized in the follow-up and management of victims, thus halting progression to bad prognosis and eventually mortality. The present study attempts to provide an in-depth analysis of different hematological and biochemical tests and associate each parameter with an enteric fever infection.

## Materials and methods

Design and study population

This study was a cross-sectional study conducted at Dschang Regional Annex Hospital, Cameroon, from November 2020 to September 2021. The study protocol was approved by the National Committee for Ethics in Human Health (CNERSH), Yaounde, Cameroon (ref: 2020/11/73/CE/CNERSH/SP). The participants were healthy individuals and those suffering from enteric fever. Male and female participants with no age restriction were selected for the study. Those included in the study were participants with or no symptoms of the disease, having signed the informed consent form and assent for those under 21. For non-inclusion criteria, the targets were those with a history of liver, kidney, and heart diseases; blood disorders; immune-compromised status; active alcohol consumption; pregnant women; positive serology for viral hepatitis; malaria; recent intake of a potential hepatotoxic drug; or any medication that may affect biochemical and hematological parameters. A total of 1082 participants were eligible for the study. Among them, subjects with hemolyzed blood serum and who did not provide complete information (n = 6) were excluded from the analysis. Finally, 1076 participants were retained for the study. Blood and stools were collected from participants who agreed to give their informed consent to participate in the study. Stool culture was used to stratify participants into sick and healthy and subdivided into 3 groups: 423 *Salmonella *Typhi positive patients, 115 *Salmonella *Paratyphi positive patients, and 538 *Salmonella *negative participants.

Stool and blood collection

Stools (25 g) were collected in wide-mouth plastic boxes from each participant and separated into two groups viz: *Salmonella* positive and negative patients. Blood (10 mL) was collected by venipuncture aseptically from the fasting participants at the elbow crease using an alcohol swab and a syringe fixed to a needle. Collected blood was separated into dry tubes to obtain serum and a tube with anticoagulant (EDTA) for evaluation of the hematological profile. The blood introduced in the dry tube was left to rest for 30 min at room temperature, then centrifuged at 3000 rpm for five minutes to obtain serum. The latter was separated from the clot into tightly screwed microfuge tubes and stored at -20 °C. These frozen sera were later analyzed for biochemical parameters.

Bacterial isolation and identification

Specimens were grown on *Salmonella Shigella* agar (SSA) and Hecktoen agar after pre-enrichment and enrichment. Then, the cultural and morphological characteristics of the isolates were studied and the isolates were duly identified following standard methods [7]. The serotyping of *Salmonella enterica *isolates was performed according to Kauffman-White Scheme [7]. Furthermore, polyvalent *Salmonella *antisera phase 1 and phase 2 flagellar H antigens were used for serovars determination of the *Salmonella* isolates.

Evaluation of biochemical and hematological parameters

Biochemical analyses such as creatinine, urea, albumin, uric acid, alkaline phosphatase, total and conjugated bilirubin, alanine aminotransferase (ALT), aspartate aminotransferase (AST), triglycerides (TG), total cholesterol, LDL cholesterol and HDL cholesterol, total protein and C-reactive protein (CRP) were run on DIALAB DTN-405 using standard methods from commercial DIALAB® Chemistry Analyzer kits (DIALAB Produktion und Vertried von chemisch-technischen Produkten Laborinstrumenten). Hematological parameters such as white blood cells (WBC), red blood cells (RBC), hemoglobin (HG), hematocrit (HTC), mean corpuscular volume (MCV), mean corpuscular hematocrit (MCH), mean corpuscular hemoglobin concentration (MCHC), platelet (PLT), lymphocytes (LYM), monocytes (MO), granulocytes (GR), red cell distribution width coefficient of variation (RDW CV), red cell distribution width standard deviation(RDW SD), mean platelet volume (MPV), platelet distribution width (PDW), and erythrocyte sedimentation rate (ESR) from whole blood were performed with a Sysmex KX-21N automated hematology analyzer (Sysmex Corporation, Kobe, Japan) using a sheath flow direct current (DC) detection method.

Statistical analysis

The biochemical and hematological data were analyzed using statistical software for the social sciences (SPSS) version 22.0 (IBM Corp., Armonk, NY). The results were expressed as mean ± standard deviation. The means of each biochemical parameter in different groups were compared using the Waller-Duncan test when significant differences were detected by ANOVA. Waller Duncan's conformity test was used to compare the means of the group variables to the reference values. Cut-off values provided by the kits were used to classify the parameter as abnormal (value out of the reference range) and normal (value within the reference range). Categorical variables were described as numbers and percentages. A Chi-square test was performed to compare the frequencies of biochemical abnormalities in the three groups. A multivariate logistic regression analysis was performed to determine true supportive parameters in enteric fever diagnosis. A p < 0.05 was considered significant.

## Results

Creatinine, urea, uric acid, alkaline phosphatase (ALP), total bilirubin, conjugated bilirubin, alanine aminotransferase (ALT), aspartate aminotransferase (AST), triglycerides, total protein, and C-reactive protein levels were found to be higher in patients with both *Salmonella *Typhi and Paratyphi compared to the control group (Table 1). On the contrary, the mean values of albuminemia, total cholesterol, LDL-cholesterol, and HDL-cholesterol were significantly decreased (p < 0.05) in *Salmonella *Typhi and Paratyphi-positive patients compared to the control group. The increased levels of uremia, uricemia, ALT, total proteins, and C-reactive protein (CRP) in positive patients with typhoid fever were higher than in those with paratyphoid fever. The mean values of all biochemical parameters in healthy participants were within the normal ranges. However, the mean values of creatininemia, uremia, albuminemia, ALP, total bilirubin, conjugated bilirubin, AST, triglycerides, total protein, C-reactive protein (in both *S.* Typhi and Paratyphi positive patients) and ALT (in solely *S.* Typhi positive patients) were out of the normal ranges.

**Table 1:** Comparison of biochemical parameters between enteric fever-positive patients and the healthy group On the same line, values affected by the different superscript letters (a, b, c) are significantly different at a p-value < 0.05 (Waller-Duncan’s test); n: number of subjects per group.

Biochemical markers	Normal values	Groups		
		S. Typhi positive patients n = 423	S. Paratyphi positive patients n = 115	S. negative participants n = 538
Creatininemia	Women (0.6 to 1 mg/dL)	1.30 ± 0.60^a^	1.29 ± 0.50^a^	0.84 ±0.31^b^
	Men (0.9 to 1.1 mg/dL)	1.34 ± 0.60^a^	1.34 ± 0.54^a^	0.98±0.23^b^
Uremia	10 to 50 mg/dL	52.42 ±1.74^a^	51.26 ± 1.35^ a^	36.59 ± 0.5^b^
Albuminemia	3.81 to 4.65 g/dL	3.06 ± 0.60^a^	3.08 ± 0.10^a^	4.10 ±1.01^b^
Uricemia	Women (2.4 to 5.7 mg/dL)	5.84 ± 1.31^a^	5.78 ±1.34^a^	4.19 ± 1.77^b^
	Men (3.4 to 7.0 mg/dL)	6.55 ± 1.40^a^	6.12 ±1.87^a^	4.35 ± 1.86^b^
Alkaline phosphatase (ALP)	(30 to 125 IU/L)	126.51 ±1.75^a^	125.76 ±3.96^a^	93.46 ±1.46^b^
Total bilirubin (TB)	0.30 to 1 mg/dL	1.07 ± 0.21^a^	1.11 ± 0.41^a^	0.71 ± 0.01^b^
Conjugated bilirubin	< 0.2 mg/dL	0.34 ± 0.07^a^	0.32 ± 0.12^a^	0.18 ± 0.04^b^
Aspartate amino-transferase (AST)	< 40 IU/L	44.01 ±0.78^a^	46.68 ± 1.34^a^	30.40 ± 0.48^b^
Alanine amino-transferase (ALT)	< 41 IU/L	51.68 ± 0.81^ a^	31.89 ± 0.61^a^	31.89 ± 0.41^b^
Total Cholesterol	< 2 g/L	0.49 ± 0.26^a^	0.45 ± 0.54^a^	1.38 ± 0.27^b^
LDL-cholesterol	< 1.3 g/L	0.46 ± 0.01^a^	0.45 ± 0.03^a^	1.05 ± 0.01^b^
HDL-cholesterol	< 0.4 g/L	0.19 ± 0.04^a^	0.20 ± 0.01^a^	0.31 ± 0.06^b^
Triglycerides	< 1.50 g/L	2.23 ±0.55^a^	1.68 ± 0.05^a^	1.48 ± 0.43^b^
Total protein	6.4 to 8.3 g/dl	8.49 ±0.83^a^	8.68 ± 0.84^a^	5.56 ± 0.69^b^
C-reactive protein (CRP)	≤ 6 mg/dl	70.83 ±4.95^a^	58.99 ±4.93^b^	4.56 ±2.8^c^

In S. Typhi and *S. *Paratyphi positive patients, the mean values of erythrocytes, mean corpuscular hemoglobin concentration (MCHC), erythrocyte sedimentation rates 1 and 2 (ESR 1 and 2) ​​were significantly (p < 0.05) increased compared to the negative group (Table 2). Nonetheless, the mean values of white blood cells (WBC), hemoglobin, hematocrit, mean corpuscular volume (MCV), mean corpuscular hemoglobin (MCH), platelet, lymphocytes, monocytes, granulocytes, red cell distribution width standard deviation (RDW SD) and plateletcrit ​​were significantly decreased (p < 0.05) in patients with both *S.* Typhi and *S.* Paratyphi compared to the control group. The mean values of all hematological parameters in *S. *Typhi and Paratyphi negative participants were within the normal ranges. The mean values of hemoglobin, mean corpuscular hemoglobin concentration (MCHC) and ESR (in *S.* Typhi and *S.* Paratyphi positive patients) are out of the normal ranges.

**Table 2:** Comparison of haematological parameters between enteric fever-positive patients and the healthy group. On the same line, values affected by the different superscript letters (a, b) are significantly different at a p-value < 0.05 (Waller-Duncan’s test); n: number of subjects per group; MCV: mean corpuscular volume; MCH: mean corpuscular hemoglobin; MCHC: mean corpuscular hemoglobin concentration; RDW CV: red cell distribution width coefficient of variation; RDW SD: red cell distribution width standard deviation; MPV: mean platelet volume; PDW: platelet distribution width; ESR: erythrocyte sedimentation rate.

Hematological markers	Normal values	Groups		
		S. Typhi-positive patients n = 423	S. Paratyphi positive patients n = 115	S. negative participants n = 538
White blood cells (WBC)	4.0 to 9.0 (10^3^/µL)	3.40 ±0.68^a^	3.28 ±0.14^a^	5.52 ± 0.10^b^
Red blood cells (RBC)	3.76 to 5.70 (10^6^/µL)	5.27±0.07^a^	5.30 ±0.14^a^	4.86 ±0.49^b^
Hemoglobin	12.0 to 18.0 (g/dL)	10.22 ±0.11^a^	10.10±0.20^a^	12.33 ±0.10^b^
Hematocrit	33.5 to 52.0 (%)	33.31 ±0.40^a^	32.88 ±0.75^a^	38.01 ±0.13^b^
MCV	80.0 to 100.0 (fL)	69.79 ±0.71^a^	69.24 ±1.44^a^	85.08 ±0.47^b^
MCH	28.0 to 32.0 (pg)	26.03 ±0.25^a^	26.47 ±0.45^a^	29.42 ±0.20^b^
MCHC	31.0 to 35.0 (g/dL)	36.11 ±0.23^a^	35.32 ±0.37^a^	32.75 ±0.22^b^
Platelet	150 to 350 (10^3^/µL)	145.18 ±3.49^a^	147.13 ± 7.93^a^	206.42 ±2.54^b^
Lymphocytes	1.0 to 4.0 (10^3^/µL)	1.63 ±0.24^a^	1.24 ±0.90^a^	2.47 ±0.69^b^
Monocytes	0.1 to 1.0 (10^3^/µL)	0.36 ±0.23^a^	0.36 ±0.38^a^	0.50 ±0.01^b^
Granulocytes	42.0 to 85.0 (%)	53.91 ±1.10^a^	49.61 ±3.13^a^	55.28±0.98^ab^
RDW CV	11.5 to 14.0 (%)	11.55 ±0.15^a^	11.50 ±0.40^a^	12.08 ±0.12^a^
RDW SD	39.0 to 46.0 (fL)	38.50 ±0.36^a^	38.67 ±0.73^a^	41.44 ±0.33^b^
Plateletcrit	0.16 to 0.33 (%)	0.19 ±0.05^a^	0.18 ±0.01^a^	0.24 ±0.05^b^
MPV	2.0 to 11.0 (f L)	8.30 ±0.69^a^	8.32 ±0.15^a^	8.18 ±1.85^a^
PDW	15.0 to 17.0 (%)	16.62 ±0.68^a^	17.02 ±0.13^a^	16.70±0.05^a^
ESR	ESR1 (3 to 7 mm/h)	34.07±1.23^a^	33.12 ±2.42^a^	5.74 ±0.10^b^
	ESR2 (7 to 12 mm/h)	62.71 ±1.15^a^	60.58 ±3.08^a^	11.64 ±0.20^b^

Of the 423 *S.* Typhi and 115 *S. *Paratyphi-positive patients, at least 60% were associated with different risk factors: cardiometabolic risk factors (hyper CRP, hyper triglyceridemia), hepatic (hyper ALP, TB, CB, AST, ALT) and renal (hyper creatininemia, hyper uremia, hyper uricemia, hypo albuminemia, hypo total protein) metabolic abnormalities (Table 3). The frequencies of hypercreatininemia, hyperuremia, hypoalbuminemia, hyper total proteinemia, hyperuricemia (renal metabolic disorders); hyper ALP, hyper total bilirubin, hyper conjugated bilirubin, hyper AST, hyper ALT (liver metabolic abnormalities); hyper triglyceridemia, and hyper C-reactive protein (cardiometabolic risk factors) were significantly (p < 0.05) higher in both *S.* Typhi and *S. *Paratyphi positive patients relative to those of negative participants (Table 3). On the contrary, the frequencies of hypo uricemia, hyper LDL-cholesterolemia, hyper HDL-cholesterolemia, hypo total proteinemia, and hyper total cholesterolemia were significantly reduced in *S.* Typhi and *S.* Paratyphi positive patients compared with those of healthy participants.

**Table 3:** Comparison of biochemical abnormalities between enteric fever-positive patients and healthy participants n: number of subjects per group.

Biochemical markers	Variations	Groups			Chi-square test (X^2^)	p-value
		S. Typhi-positive patients n = 423	S. Paratyphi-positive patients n = 115	S. negative participants n = 538		
Creatininemia	Hypo	55 (13.0%)	11(9.6%)	82 (15.2%)	464.270	0.000
	Hyper	287(67.8%)	88 (76.5%)	43 (8.0%)		
	Normal	81 (19.1%)	16 (13.9%)	413 (76.8%)		
Uremia	Hypo	45 (10.6%)	14 (12.2%)	60 (11.2%)	272.650	0.000
	Hyper	269(63.6%)	70(60.9%)	85 (15.8%)		
	Normal	109 (25.8%)	31 (27.0%)	60(11.2%)		
Albuminemia	Hypo	350 (82.7%)	90 (78.3%)	87 (16.2%)	507.951	0.000
	Hyper	35(8.53%)	17 (14.8%)	67 (12.5%)		
	Normal	38 (9.0%)	8 (7.0%)	384 (71.4%)		
Uricemia	Hypo	14 (3.3%)	7 (6.1%)	110 (20.4%)	287.812	0.000
	Hyper	300 (70.9%)	81 (70.4%)	108 (20.1%)		
	Normal	109 (25.8%)	27 (23.5%)	320 (59.5%)		
Alkaline Phosphatase (ALP)	Hypo	31(7.3%)	12(10.4%)	26 (4.8%)	252.455	0.000
	Hyper	264(62.4%)	69 (60.0%)	94 (17.5%)		
	Normal	128(30.3%)	34(29.6%)	418(77.7%)		
Total bilirubin (T B)	Hypo	31 (7.3%)	12(10.4%)	57 (10.6%)	304.933	0.000
	Hyper	279 (66.0%)	78(67.8%)	81(15.1%)		
	Normal	113(26.7%)	25 (21.7%)	400(74.3%)		
Conjugated bilirubin (CB)	Hyper	320(75.7%)	89 (77.4%)	118 (21.9%)	315.041	0.000
	Normal	103(24.3%)	26(22.6%)	420(78.1%)		
Aspartate aminotransférase (AST)	Hyper	322 (76.1%)	88 (76.5%)	80(14.9%)	408.087	0.000
	Normal	101 (23.9%)	27(23.5%)	458(85.1%)		
Alanine aminotransférase (ALT)	Hyper	335 (79.2%)	88 (76.5%)	70 (13.0%)	466.755	0.000
	Normal	88^a^ (20.8%)	27(23.5%)	468(87.0%)		
Total cholesterol	Hyper	12 (2.8%)	5(4.3%)	59 (11.0%)	25.289	0.000
	Normal	411 (97.2%)	110(95.7%)	479(89.0%)		
LDL-cholesterol	Hyper	18(4.3%)	8(7.0%)	85(15.8%)	35.681	0.000
	Normal	405(95.7%)	107 (93.0%)	453 (84.2%)		
HDL-cholesterol	Hyper	16 (3.8%)	1(0.9%)	108 (20.1%)	75.703	0.000
	Normal	407 (96.2%)	114 (99.1%)	430(79.9%)		
Triglycerides	Hypo	4(0.9%)	2(1.7%)	0(0.0%)	330.888	0.000
	Hyper	285 (67.4%)	70(60.5%)	70(13.0%)		
	Normal	134 (31.7%)	43 (47.4%)	468 (87.0%)		
Total proteins	Hypo	30(7.1%)	7 (6.1% )	304(56.5%)	685.145	0.000
	Hyper	330(78.0%)	96(83.5%)	9(1.7%)		
	Normal	63(14.9%)	12(10.4%)	225(41.8%)		
C-reactive protein (CRP)	Hyper	409(97.7%)	111(96.5%)	226(42.0%)	229.313	0.000
	Normal	14 (3.3%)	4(3.5%)	312 (58.0%)		

The frequencies of low WBC, low lymphocytes, low monocytes, low granulocytes, high RBC, decreased hemoglobin level, decreased hematocrit, low MCV, low MCH, increased MCHC level, decreased platelet, as well as increased PDW, ESR1, and ESR2 levels, were observed in the enteric fever positive patients when compared with those of healthy individuals (Table 4).

**Table 4:** Comparison of hematological abnormalities between enteric fever-positive patients and healthy participants CI: confidence interval; n: number of subjects per group; MCV: mean corpuscular volume; MCH: mean corpuscular hemoglobin; MCHC: mean corpuscular hemoglobin concentration; RDW CV: red cell distribution width coefficient of variation; RDW SD: red cell distribution width standard deviation; MPV: mean platelet volume; PDW: platelet distribution width; ESR: erythrocyte sedimentation rate.

Hematological parameters	Variations	Groups			Chi-square test (X^2^)	p-value
		S. Typhi-positive patients n = 423	S. Paratyphi-positive patients n = 115	S. negative participants n = 538		
White blood cells (WBC)	Hypo	349 (82.5%)	101 (87.8%)	160(29.7%)	323.161	0.000
	Hyper	3 (0.7%)	3 (2.6%)	57 (10.6%)		
	Normal	71 (16.8%)	11 (9.6%)	311 (59.7%)		
Red blood cells (RBC)	Hypo	49 (11.6%)	12 (10.4%)	78 (14.5%)	18.393	0.000
	Hyper	118 (27.9%)	34 (29.6%)	94 (17.5%)		
	Normal	256 (60.5%)	69 (60.0%)	366 (68.0%)		
Hemoglobin	Hypo	362 (85.6%)	97 (84.3%)	204 (37.9%)	275.521	0.000
	Hyper	0 (0.0%)	2 (1.7%)	0 (0.0%)		
	Normal	61 (14.4%)	16 (13.9%)	334 (62.1%)		
Hematocrit	Hypo	267 (63.1%)	75 (65.2%)	179 (33.3%)	99.193	0.000
	Hyper	12 (2.8%)	3 (2.6%)	24 (4.5%)		
	Normal	144 (34.9%)	37 (32.2%)	335 (62.5%)		
MCV	Hypo	323 (76.4%)	87 (75.7%)	165 (30.2%)	232.176	0.00
	Hyper	7 (1.7%)	1 (0.9%)	8 (1.5%)		
	Normal	93 (22.0%)	27 (23.5%)	367 (68.2%)		
MCH	Hypo	283 (66.9%)	65 (56.5%)	181 (33.6%)	141.392	0.000
	Hyper	53 (12.5%)	9 (7.8%)	45 (8.4%)		
	Normal	87 (20.6%)	41 (35.7%)	312 (58.0%)		
MCHC	Hypo	73 (17.3%)	15 (13.0%)	127 (23.6%)	257.010	0.000
	Hyper	329 (77.8%)	95 (82.6%)	187 (34.8%)		
	Normal	73 (17.3%)	17 (14.7%)	224 (41.6%)		
Platelet	Hypo	291 (68.8%)	84 (73.0%)	179 (33.3%)	159.590	0.000
	Hyper	21 (5.0%)	4 (3.5%)	17 (3.2%)		
	Normal	111 (26.2%)	27 (23.5%)	342 (63.6%)		
Lymphocytes	Hypo	221 (55.2%)	63 (54.8%)	103 (19.1%)	152.902	0.000
	Hyper	22 (5.2%)	5 (4.3%)	94 (17.5%)		
	Normal	180 (42.6%)	47 (40.9%)	341 (63.4%)		
Monocytes	Hypo	202 (47.8%)	56 (48.7%)	121 (22.5%)	77.546	0.000
	Hyper	53 (12.5%)	12 (10.4%)	85 (15.8%)		
	Normal	168 (39.7%)	47 (40.9%)	332 (61.7%)		
Granulocytes	Hypo	203 (48.0%)	55 (47.8%)	179 (33.3%)	25.341	0.000
	Hyper	57 (13.5%)	11 (9.6%)	88 (16.4%)		
	Normal	163 (38.5%)	49^b^ (42.6%)	271 (50.4%)		
RDW CV	Hypo	226 (53.4%)	77 (67.0%)	219 (40.7%)	33.251	0.000
	Hyper	47 (11.1%)	9 (7.8%)	69(12.9%)		
	Normal	150 (35.5%)	29 (25.2%)	250 (46.5%)		
RDW SD	Hypo	221 (52.2%)	67 (58.3%)	160 (29.7%)	64.122	0.000
	Hyper	50 (11.8%)	11 (9.6%)	89 (16.5%)		
	Normal	152 (35.5%)	37 (32.2%)	289 (53.7%)		
Plateletcrit	Hypo	215 (50.8%)	56 (48.7%)	109 (20.3%)	107.928	0.000
	Hyper	3 (8.7%)	8 (7.0%)	82 (15.6%)		
	Normal	171 (40.4%)	51 (44.3%)	347 (64.5%)		
MPV	Hypo	0 (0.0%)	0 (0.0%)	2 (0.4%)	3.612	0.461
	Hyper	11 (2.6%)	4 (3.5%)	22 (4.1%)		
	Normal	412 (97.4%)	111(96.5%)	514 (95.5%)		
PDW	Hypo	52 (12.3%)	16 (13.9%)	38 (7.1%)	28.581	0.000
	Hyper	206 (48.7%)	62 (53.9%)	218 (40.5%)		
	Normal	165 (38.0%)	37 (32.2%)	282 (52.4%)		
ESR1	Hypo	0 (0.0%)	0 (0.0%)	2 (0.4%)	684.565	0.000
	Hyper	393 (92.9%)	109 (94.8%)	74 (13.8%)		
	Normal	30 (7.1%)	6 (5.2%)	462 (85.9%)		
ESR2	Hyper	412 (97.4%)	111 (96.5%)	111 (20.4%)	654.522	0.000
	Normal	11 (2.6%)	4 (3.5%)	428 (79.6%)		

Results of multiple logistic regression analysis showed positive and significant associations (OR > 1; p < 0.05) between *S. enterica* (serovars Typhi and Paratyphi) infection and hyper creatininemia, hypoalbuminemia, hyper ALP, hyper total bilirubinemia, hyper conjugated bilirubinemia, hyper ALT, hyper triglyceridemia, hyper total proteinemia, hyper CRP (Table 5). Similarly, *S.* Paratyphi infection was significantly associated with hypo triglyceridemia while hyper AST was an independent predictor of *S.* Typhi infection.

**Table 5:** Association of biochemical parameters with enteric fever infection following multivariate logistic regression analysis. Ref. : reference category; /: none

Biochemical Parameters	Variation	S. Typhi infection		S. Paratyphi infection	
		Odds Ratio (95% CI)	p-value	Odds Ratio (95% CI)	p-value
Creatininemia	Hypo	8.199 (1.201-55.957)	0.032	7.556 (0.978-58.390)	0.053
	Hyper	15.890 (3.389-74.494)	0.000	22.485 (4.456-113.461)	0.000
	Normal	Ref.	/	Ref.	/
Uremia	Hypo	2.348 (0.364-13.897)	0.384	2.243 (0.0330-15.241)	0.409
	Hyper	2.808 (0.546-14.436)	0.216	2.349 (0.435-12.695)	0.321
	Normal	Ref.	/	Ref.	/
Albuminemia	Hypo	29.056 (5.461-154.601)	0.000	33.601 (5.536-203.956)	0.000
	Hyper	4.283 (0.502-36.573)	0.184	9.621 (0.984-94.056)	0.052
	Normal	Ref.	/	Ref.	/
Uricemia	Hypo	0.735 (0.076-7.118)	0.735	1.600 (0.146-17.528)	0.700
	Hyper	3.853 (0.891-16.660)	0.071	4.464 (0.976-94.056)	0.054
	Normal	Ref.	/	Ref.	/
Alkaline phosphatase (ALP)	Hypo	2.133 (0.170-26.701)	0.557	3.021 (0.224-40.762)	0.405
	Hyper	5.927 (1.391-25.250)	0.016	5.840 (1.305-26.141)	0.021
	Normal	Ref.	/	Ref.	/
Total bilirubin	Hypo	35.480 (3.874-423.982)	0.02	62.018 (6.177-622.685)	0.000
	Hyper	23.819 (3.895-145.668)	0.01	31.208 (4.862-200.318)	0.000
	Normal	Ref.		Ref.	/
Conjugated bilirubin	Hyper	6.537 (1.599-26.715)	0.009	6.467 (1.490-200.318)	0.013
	Normal	Ref.	/	Ref.	/
Aspartate aminotransferase (AST)	Hyper	3.350 (0.796-14.093)	0.009	3.334 (0.751-14.894)	0.113
	Normal	Ref.	/	Ref.	/
Alanine aminotransferase (ALT)	Hyper	18.400 (4.332-80.002)	0.000	15.433 (3.358-70.936)	0.000
	Normal	Ref.	/	Ref.	/
Total cholesterol	Hyper	1.012 (0.064-16.053)	0.993	1.970 (0.109-35.582)	0.646
	Normal	Ref.	/	Ref.	/
LDL-cholesterol	Hyper	0.254 (0.021-3.006)	0.277	0.515 (0.040-6.580)	0.610
	Normal	Ref.	/	Ref.	/
HDL-cholesterol	Hyper	0.276 (0.035-2.173)	0.221	0.059 (0.003-1.000)	0.050
	Normal	Ref.	/	Ref.	/
Triglycerides	Hypo	53.009 (8.390-98.908)	0.600	68.860 (10.998-431.131)	0.000
	Hyper	17.925 (3.887-82.654)	0.000	14.332 (2.984-68.842)	0.001
	Normal	Ref.	/	Ref.	/
Total proteins	Hypo	0.421 (0.078-2.277)	0.315	0.497 (0.075-3.294)	0.469
	Hyper	44.703 (6.250-319.739)	0.000	69.332 (8.960-536.483)	0.000
	Normal	Ref.	/	Ref.	/
C- reactive protein (CRP)	Hyper	9.634 (0.736-127.120)	0.025	8.430 (0.541-131.241)	0.028
	Normal	Ref.	/	Ref.	/

Regarding the association between hematological parameters and enteric fever infection, positive and significant (OR > 1; p < 0.05) relationships between *S.* *enterica *(serovars Typhi and Paratyphi) infection, and leukopenia, thrombocytopenia, lymphopenia, monocytopenia, low hemoglobin, low hematocrit, low MCV, low MCH, low platelet, low plateletcrit, high PDW, high ESR1 were observed (Table 6). Similarly, hyper ESR2 was an independent predictor of *S. *Typhi infection. However, a negative and significant association (OR < 1; p < 0.05) was recorded between *S. *enterica (serovars Typhi and Paratyphi) infection and high MPV.

**Table 6:** Association of hematological parameters with enteric fever infection following multivariate logistic regression analysis. Ref. : reference category; /: none; CI: confidence interval; MCV: mean corpuscular volume; MCH: mean corpuscular hemoglobin; MCHC: mean corpuscular hemoglobin concentration; RDW CV: red cell distribution width coefficient of variation; RDW SD: red cell distribution width standard deviation; MPV: mean platelet volume; PDW: platelet distribution width; ESR: erythrocyte sedimentation rate.

Hematological Parameters	Variation	S. Typhi infection		S. Paratyphi infection	
		Odds Ratio (95% CI)	p-value	Odds Ratio (95% CI)	p-value
White blood cells (WBC)	Hypo	8.076 (3.284-19.860)	0.000	15.440 (5.246-45.443)	0.000
	Hyper	0.545 (0.047-6.332)	0.627	3.582 (0.273-46.922)	0.331
	Normal	Ref.	/	Ref.	/
Red blood cells (RBC)	Hypo	2.006 (0.601-6.695)	0.257	1.900 (0.501-7.199)	0.345
	Hyper	2.326 (0.866-6.245)	0.094	2.352 (0.814-6.796)	0.114
	Normal	Ref.	/	Ref.	/
Hemoglobin	Hypo	6.541 (2.566-16.673)	0.000	6.900 (2.378-20.016)	0.000
	Hyper	0.524 (0.892-6.758)	0.0721	0.790 (1.285-9.103)	0.621
	Normal	Ref.	/	Ref.	/
Hematocrit	Hypo	2.761 (1.158-6.583)	0.022	3.198 (1.244-8.224)	0.016
	Hyper	0.662 (0.074-5.895)	0.712	0.658 (0.055-7.832)	0.741
	Normal	Ref.	/	Ref.	/
MCV	Hypo	12.821 (5.236-31.395)	0.000	11.005 (4.119-29.400)	0.000
	Hyper	8.082 (0.651-100.366)	0.104	3.971 (0.153-102.822)	0.406
	Normal	Ref.	/	Ref.	/
MCH	Hypo	5.259 (2.124-13.021)	0.000	2.685 (1.008-7.149)	0.048
	Hyper	4.241 (1.173-15.333)	0.028	1.619 (0.380-6.889)	0.515
	Normal	Ref.	/	Ref.	/
MCHC	Hypo	10.218 (2.535-41.191)	0.001	6.682 (1.226-36.421)	0.028
	Hyper	22.265 (6.043-82.032)	0.000	22.071 (4.651-104.746)	0.000
	Normal	Ref.	/	Ref.	/
Platelet	Hypo	3.362 (1.440-7.851)	0.005	4.108 (1.600-10.547)	0.003
	Hyper	6.989 (0.822-59.423)	0.075	4.921 (0.463-52.296)	0.186
	Normal	Ref.	/	Ref.	/
Lymphocytes	Hypo	5.970 (2.390-14.748)	0.000	6.831 (2.554-18.265)	0.000
	Hyper	0.615 (0.139-2.720)	0.522	0.910 (0.160-5.178)	0.915
	Normal	Ref.	/	Ref.	/
Monocytes	Hypo	11.273 (4.003-31.748)	0.000	10.464 (3.479-31.478)	0.000
	Hyper	0.562 (0.166-1.905)	0.355	0.379 (0.096-1.497)	0.166
	Normal	Ref.	/	Ref.	/
Granulocytes	Hypo	1.882 (0.758-4.674)	0.173	1.805 (0.679-4.800)	0.237
	Hyper	2.872 (0.812-10.153)	0.102	2.015 (0.497-8.162)	0.326
	Normal	Ref.	/	Ref.	/
RDW CV	Hypo	1.701 (0.718-4.030)	0.227	2.684 (1.036-6.952)	0.042
	Hyper	2.168 (0.515-9.136)	0.292	2.597 (0.517-13.037)	0.246
	Normal	Ref.	/	Ref.	/
RDW SD	Hypo	1.716 (0.706-4.169)	0.233	1.745 (0.665-4.575)	0.258
	Hyper	0.077(0.222-2.669)	0.681	0.584 (0.142-2.400)	0.456
	Normal	Ref.	/	Ref.	/
Plateletcrit	Hypo	4.412 (1.600-12.168)	0.004	3.718 (1.268-10.901)	0.017
	Hyper	0.569 (0.159-2.031)	0.385	0.355 (0.081-1.563)	0.171
	Normal	Ref.	/	Ref.	/
MPV	Hypo	0.008 (0.000-0.1)	0.996	0.024 (0.140-3.201)	0.998
	Hyper	0.023 (0.004-0.138)	0.000	0.029 (0.004-0.214)	0.001
	Normal	Ref.	/	Ref.	/
PDW	Hypo	2.000 (0.403-9.925)	0.396	3.392 (0.622-18.507)	0.158
	Hyper	2.613 (1.079-6.325)	0.033	3.673 (1.395-9.668)	0.008
	Normal	Ref.	/	Ref.	/
ESR1	Hypo	3.444 (0.901-9.731)	0.991	9.346 (2.504-23.492)	0.996
	Hyper	4.890 (4.413-50.240)	0.000	33.891 (6.163-186.337)	0.000
	Normal	Ref.	/	Ref.	/
ESR2	Hyper	15.467 (4.072-58.756)	0.000	5.701 (0.829-39.194)	0.077
	Normal	Ref.	/	Ref.	/

## Discussion

Enteric fever is a systemic disease that affects multiple organs of the body. The liver, kidney, and cardiac systems are essential for the proper functioning of the body. Hence, damage to these organs affects the health of people with the disease. In the present study, the results of the multiple logistic regression analysis showed positive and significant associations (OR > 1; p < 0.05) between enteric fever and hyper creatininemia, hypoalbuminemia, hyper ALP, hyper total bilirubin, hyper conjugated bilirubin, hyper ALT, hyper triglyceridemia, hyper total proteinemia, hyper CRP; indicating that these biochemical abnormalities could act as diagnostic factors for enteric fever. Moreover, they may also be crucial in the correct management of patients and in predicting disease prognosis. Similarly, *S.* Paratyphi infection was significantly associated with hypo triglyceridemia while hyper AST was observed as an independent predictor of *S.* Typhi infection.

Renal involvement is an uncommon event during enteric fever, occurring only in 2-3% of patients [8]. Urea is known to be a nitrogenous compound formed in the liver as an end product of protein catabolism and about 85% of urea is eliminated by the kidneys and the rest is excreted through the gastrointestinal tract. Creatinine, a byproduct of creatine phosphate in the muscle is entirely eliminated by the kidney. A simultaneous increase in creatinine and urea in most patients with enteric fever indicates renal dysfunction. This can be linked to an increased catabolic rate which characterizes the disease. In addition, renal impairment can be explained by immune complex deposits and toxin-induced nephropathy, which is a direct result of the toxin effect on the podocytes [9]. The raised urea and creatinine levels in enteric fever have been reported [10]. On the contrary, urea was observed to be non-affected in patients with enteric fever in another study [11].

The liver is commonly implicated in patients with typhoid and paratyphoid fevers. In this study, we observed hyper alkaline phosphatase (62.4%), hyper ALT (79.2%), and hyper total bilirubin (66.0%), which are quite higher than those already reported: 44%, 73.3%, 30.6%, respectively [12]. A previous study conducted in India showed a significant increase in the levels of ALT, AST, PAL, total and conjugated bilirubin as well as a significant decrease in albumin levels [13]. Increased hepatic enzymes is suggestive of liver damage. This can be due to specific endotoxin effects locally or systemically, inflammatory reactions to ulceration of intestines, and *S.* Typhi cytotoxin production in infected Kupffer cells or liver macrophages [14]. High total bilirubin (jaundice) in enteric fever patients is a result of hemolysis and in severe conditions can lead to liver injury. Additionally, hyper-bilirubinemia observed in patients with enteric fever could be due to the multiplication of *S.* Typhi/Paratyphi within the biliary tract which causes clogging of the biliary flow [15]. The incidence of jaundice and raised bilirubin levels in enteric fever have been reported [14]. In the current investigation, fever and jaundice were the presenting symptoms in 66.0% which is quite higher than that obtained by earlier investigators (30.6%) [12]. Our results demonstrate that liver involvement in enteric fever is usually associated with extrahepatic complications. Generally, hypoproteinemia is known to be associated with complications and mortality in patients with acute infectious disease [16]. Albumin and total proteins were also significantly lower in enteric fever patients compared to normal individuals. This result disagrees with the findings of earlier investigators who showed an increase in albumin in chronic typhoid patients [17]. Nevertheless, suggestive mechanisms for the drop of plasma proteins have been proposed thus: large vascular leakage of plasma proteins owing to raised capillary permeability, reduced protein intestinal absorption due to low ingestion, albumin clearance in urine, elevated protein catabolism, and diminished liver synthesis due to hepatic damage [18]. Additionally, proteins can be lost as a result of kidney dysfunctions which lead to an excessive amount of proteins being excreted in the urine.

The lipid profile is known to alter in patients with severe sepsis; hence, the evaluation of the lipid profile could be necessary for performing a typhoid fever diagnosis. In this study, patients with enteric fever showed a significant increase in the level of triglycerides compared to their controls. This finding agreed with that of a previous study which showed an elevation in the levels of triglycerides in patients with enteric fever [19]. This was unlike another study that showed hypocholesterolemia and hyper LDL-cholesterolemia as well as a non-significant change in triglyceride and HDL-cholesterol levels in typhoid fever [20]. This disparity in results may be due to differences in analytic methods used and the extent of illness in a patient as well as the patient’s dietary intake. The increase in triglyceride levels can be explained by increased biosynthesis during infection [21]. High levels of triglycerides in the bloodstream have been linked to atherosclerosis which increases the risk of heart disease and stroke.

Although the C-reactive protein (CRP) test is non-specific, it is very useful in the characterization of acute inflammation and infection [22]. The inflammatory conditions and host responses to infection cause the release of interleukin-6 and other cytokines that trigger the synthesis of CRP and fibrinogen by the liver [22]. Hence, we suggest using CRP in enteric fever detection given the significant and positive relationship (OR > 1; p < 0.05) between high levels of CRP and enteric fever.

The results of our investigation also revealed positive and significant (OR > 1; p < 0.05) relationships between enteric fever infection and leukopenia, thrombocytopenia, lymphopenia, monocytopenia, low hemoglobin, hematocrit, mean corpuscular volume (MCV), mean corpuscular hemoglobin (MCH) and plateletcrit levels as well as high platelet distribution width (PDW) and erythrocyte sedimentation rate 1 (ESR1) levels. Similarly, higher ESR2 was an independent predictor of *S. *Typhi infection. Nonetheless, a negative and significant association (OR < 1; p < 0.05) was recorded between enteric fever infection and high mean platelet volume (MPV). This observation is indicative that hematological abnormalities can reinforce a positive enteric fever condition and also contribute to disease management. Enteric fever is a multisystem disease that activates bone marrow which results in an increase in MPV, PDW, and ESR. ESR measures non-specific inflammation. The significant increase in ESR in infected patients may be due to an increased inflammatory response. The decrease in the levels of some hematological parameters (WBC, platelet, lymphocytes, monocytes, hemoglobin, hematocrit, MCV, MCH, and plateletcrit) may be a result of declined bone marrow activity and hemophagocytosis. The latter is the main route of elimination of the pathogen in victims of enteric fever [23]. Our results are consistent with those of a previous report which showed that most patients with typhoid fever are moderately anemic, and have leukopenia, thrombocytopenia, and relative lymphocytopenia [23]. Thrombocytopenia was reported as the most common complication of enteric fever [23] whereas leukopenia is considered a common hematological finding in enteric fever [12].

In this study, leukopenia was observed at least in 82.5% of cases, which is quite higher than the 11.2% previously reported [24]. Leucocytosis in children is common within 10 days of sickness and in those experiencing bleeding [25]. However, in the current study, leucocytosis was seen in less than 3% of patients with enteric fever. The low hematocrit and hemoglobin levels recorded in patients with typhoid/paratyphoid fever indicate that anemia could be involved in at least 63.0% of patients. A previous study documented anemia in 48.0% of patients with enteric fever [26]. Hemolysis, elements of gastrointestinal blood loss, and transient marrow suppression have been cited as the mechanisms of anemia during enteric fever [24]. Low MCV means that red blood cells (RBC) are smaller than normal and may indicate microcytic anemia. This condition may be caused by iron deficiency. The mean corpuscular hemoglobin concentration (MCHC) levels were abnormally low in the majority of the patients, suggesting microcytic hypochromic anemia. This result is discordant with those of an earlier study which showed that enteric fever was associated with normocytic normochromic anemia [27]. The hematological and biochemical changes observed in this study can be elucidated by multiple organ implications in enteric fever with hallmarks of bacteremia and sustained normal fever. Few studies have been performed on hematological and biochemical profiles of enteric fever patients in Cameroon, but no study report is available yet on hematological profiles in our region.

## Conclusions

Overall, our results reveal high elevation and diminution of certain biological factors which could be exploited for healthcare benefits in conditions of enteric fever. Our study shows that enteric fever is associated with hyper creatininemia, hypoalbuminemia, hyper total proteinemia, hyper ALP, hyper total bilirubinemia, hyper conjugated bilirubin, hyper ALT, hyper triglyceridemia, hyper CRP, leukopenia, thrombocytopenia, lymphopenia, monocytopenia, low plateletcrit, high platelet distribution width (PDW) level, and high erythrocyte sedimentation rate. Moreover, this study brings to light a high prevalence of microcytic hypochromic anemia in the majority of patients with enteric fever. Our findings emphasize the importance of using these biochemical and hematological markers to enhance serological diagnosis and for the appropriate management of enteric fever patients.
